# PRENACEL partner - use of short message service (SMS) to encourage male involvement in prenatal care: a cluster randomized trial

**DOI:** 10.1186/s12978-020-0859-6

**Published:** 2020-04-06

**Authors:** Lívia Pimenta Bonifácio, Ana Carolina Arruda Franzon, Fabiani Spessoto Zaratini, Fernanda Bergamini Vicentine, Francisco Barbosa-Júnior, Giordana Campos Braga, Jazmin Andrea Cifuentes Sanchez, Lívia Oliveira-Ciabati, Magna Santos Andrade, Mariana Fernandes, Suzi Volpato Fabio, Geraldo Duarte, Vicky Nogueira Pileggi, João Paulo Souza, Elisabeth Meloni Vieira

**Affiliations:** 1 Department of Social Medicine, Ribeirao Preto Medical School, University of São Paulo, Ribeirao Preto, São Paulo, Brazil; 2 Department of Gynecology and Obstetrics, Ribeirao Preto Medical School, University of São Paulo, Ribeirao Preto, São Paulo, Brazil; 3 Department of Education, College of Nursing, State University of Bahia (UNEB), Senhor do Bonfim, Bahia, Brazil; 4 Women Health Programme, Ribeirao Preto Health Department, Ribeirao Preto, São Paulo, Brazil

**Keywords:** Paternal involvement, Prenatal, mHealth, Text messaging, SMS text, envolvimento paterno, pré-natal, mHealth, mensagens de texto, SMS

## Abstract

**Background:**

The partner has an important role when he participates of the prenatal care as showed in the positive results relate to the mother and the child health. For this reason it is an important strategy to bring future fathers closer to health services and to improve their link with paternity.

**Aim:**

To evaluate whether the implementation of SMS technology, through the PRENACEL program for the partner as a health education program, is a useful supplement to the standard prenatal monitoring.

**Methods:**

A parallel cluster randomized trial was carried out, with the clusters representing primary care health units. The 20 health units with the largest number of pregnant women in 2013 were selected for the study. There was a balance of the health units according to the size of the affiliated population and the vulnerability situation and these were allocated in intervention and control health units by the randomization. The partners of the pregnant women who started prenatal care prior to the 20th week of gestation were the study population of the intervention group. The participants received periodic short text messages via mobile phone with information about the pregnancy and birth. In the control group units the partners, together with the women, received the standard prenatal care.

**Results:**

One hundred eighty-six partners were interviewed, 62 from the PRENACEL group, 73 from the intervention group that did not opt ​​for PRENACEL and 51 from the control group. A profile with a mean age of 30 years was found and the majority of respondents (51.3%) declared themselves as brown race/color. The interviewees presented a mean of 9.3 years of study. The majority of the men (95.2%) cohabited with their partner and 63.7% were classified as socioeconomic class C. The adherence to the PRENACEL program was 53.4%. In relation to the individual results, there was a greater participation of the PRENACEL partners in the prenatal consultations, as well as a greater presence of them accompanying the woman at the moment of the childbirth when compared to the other groups.

**Conclusion:**

The study showed that a health education strategy using communication technology seems to be a useful prenatal care supplement; the intervention had a good acceptability and has a promising role in men’s involvement in prenatal, labour and postpartum care of their partners.

**Trial registration:**

Clinical trial registry: RBR-54zf73, U1111–1163-7761.

## RESUMO

**Introdução:**

O parceiro tem um papel importante quando ele acompanha o pré-natal, parto e pós-parto de sua companheira como mostram os resultados positivos em relação à saúde materna, infantil e também relacionados à saúde do homem. É uma importante estratégia de aproximar os futuros pais dos serviços de saúde e melhorar o vínculo destes com a paternidade.

**Objetivo:**

Avaliar se a implementação da tecnologia SMS através do programa PRENACEL para o parceiro como um programa de educação em saúde é um suplemento útil ao acompanhamento pré-natal padrão.

**Método:**

Ensaio aleatorizado controlado por conglomerados representados por unidades de saúde de atenção primária. Selecionamos as 20 unidades de saúde com maior número de gestantes acompanhadas em 2013. Segundo critérios de tamanho da população adscrita e pela situação de vulnerabilidade houve um balanço e as unidades de saúde foram aleatoriamente alocadas em unidades intervenção e controle. Os parceiros das gestantes que iniciaram o pré-natal antes da 20ª semana de gestação foram a população do grupo intervenção. Os parceiros inscritos no PRENACEL receberam periodicamente mensagens curtas de texto via celular com informações sobre gestação e parto. Nas unidades do grupo controle os parceiros receberam, junto com suas companheiras, o pré-natal padrão.

**Resultados:**

186 parceiros foram entrevistados, 62 do grupo PRENACEL, 73 do grupo intervenção, mas que não optaram pelo PRENACEL e 51 do grupo controle. Encontramos um perfil com idade média de 30 anos e a maioria dos entrevistados (51%) se declarou como raça/cor parda. Os entrevistados tinham em média de 9,3 anos de estudo. A maioria dos homens (95,2%) coabita com a companheira e foi classificada como classe C (63,7%). A adesão ao programa PRENACEL foi de 53,4%. Em relação aos resultados individuais, houve uma maior participação dos parceiros do grupo PRENACEL nas consultas de pré-natal, assim como foi observada uma maior presença destes no momento do parto como acompanhante quando comparado aos demais grupos.

**Conclusão:**

O estudo mostrou que uma estratégia de educação em saúde utilizando as tecnologias de comunicação parece ser um suplemento útil para o cuidado pré-natal, teve uma boa aceitabilidade e um papel promissor no engajamento de homens aos cuidados pré-natal, parto e pós-parto de suas companheiras.

## Plain English summary

There are data of the positive results of involvement the partners in relation to the maternal health, since it can improve the quality of the health treatment of pregnant women and help to reduce the maternal mortality ratio, being an important challenge in some countries as in Brazil. The use of the mobile technology called mHealth is a new and attractive intervention to provide safe health information to users by using cell phone. Associate the use of this technology could increase the involvement of men in the maternal aspects, therefore, improve the maternal outcomes, strengthen the couple’s responsibility with the future child and bring men closer to fatherhood. This study assessed if the implementation of a program called PRENACEL that transmit information using messages (SMS) to pregnant women’s partner would be a useful supplement for the prenatal care. We compared three groups, men who received the encourage and education messages (SMS – PRENACEL group), men who were of the same health units but choose not participated of the PRENACEL (non-PRENACEL group) and the Control group composed of men who did not receive the messages and who belonged to the control health units with standard prenatal care.. The study showed that the men who received the messages attended more prenatal consultations with their pregnant partners and was also the most present group at the time of birth, compared with two other groups. The intervention of the PRENACEL program seemed to be a good strategy to motivate the partners to participate of the maternal context.

## Background

Maternal mortality ratio (MMR) is an important health and social development indicator. But despite advances in reducing maternal mortality worldwide and progress made in Brazil, in 2016, the Sustainable Development Goals stipulated a new set of goals and the target of reducing the global MMR to less than 70 deaths per 100,000 live births. The target MMR for Brazil is to achieve 20 deaths per 100,000 livebirths by 2030. Besides the MMR reduction, the goal also include improving the quality and access of health services offered to pregnant women and integrating the family into reproductive health care [1–7].

According to The International Childbirth Initiative, a publication by the FIGO Safe Motherhood and Newborn Health Committee, one of the 12 steps to safe and respectful motherhood mentions the presence and support of a partner or husband as important in favoring the care of women and babies in this period [8]. As well as other papers in the literature show there are benefits of partner support during pregnancy, prenatal care (PNC) consultations, labour, childbirth and postpartum.

Several authors have studied the role and presence of husband / partner in PNC, childbirth and postpartum consultations. The partner’s role has been mainly to be a trusted person and a social support for the pregnant woman. Positive results were observed, as well as in the health of the pregnant woman’s own partner. This shows that bringing the closest partner to health services favors self-care and the identification and treatment of diseases in the male population [9, 10]. The presence of the partner has also encouraged the increase in women’s knowledge of care during this period, the demand for qualified and appropriate care during childbirth and the early pursuit of postnatal care, resulting in better baby conditions [11–16].

Monguilhott et al. (2018) presented that the presence of the partner at the time of labour was associated with increased supply of hydration and food to the pregnant woman, prescription of a specific diet, use of non-pharmacological pain relief measures, adoption of different postures during labour and favored skin-to-skin contact, as well as reduced the use of enema, trichotomy, and Kristeller’s maneuver during labour [17].

Women who have support during the labour from a companion chosen by them are more satisfied with the care they receive, with the medical guidance and with the general experience [18]. The involvement and the presence of the partner in the PNC and especially in the childbirth is a factor of protection for the woman and the baby [9, 19].

The study by Hildingsson et al. (2011) showed that many men have a strong desire to participate in the childbirth and share this experience with their partners. However, many feel unprepared to assist in the labour, since they do not have knowledge or information [20]. In addition, there are institutional barriers that can discourage the participation of the man [21–23].

Providing more accessible information and knowledge base would be a way to stimulate men’s involvement in health care, address some of these barriers and to be more encouraged to participate. One way to provide more information and knowledge is to use communication technology tools. The WHO defines eHealth as the use of information and communication technologies for health, also found in the literature as telemedicine, SMS and mHealth [24]. The use of the technological resources of communication in health is a promising, innovative field and is being widely studied, due to being strategies that arouse curiosity and generate greater adherence in men [25, 26].

Reviews of studies using mHealth technology for maternal and newborn health have shown positive results in improving quality of care with increased demand for health services, which means more consultations, better care, proper labour and care with vaccinations [27–30].

Kumar et al. (2008) showed that educational interventions with the couple presented more satisfactory results in the target behavior related to maternal health when compared to educational work focused only on women [31]. This reinforces the context of health education; better-informed couples are more likely to have healthier health behaviors, as better-informed men are more likely to participate more in a couple’s decisions to have children and when to have them and to become more involved in family and care issues [32–35].

The study by Bruggemann et al. (2016) showed that strengthening health education strategies, especially in prenatal care, for pregnant women and their partners favors the claim for users’ rights in favor of quality care, empowering the couple through knowledge.

Considering that the dissemination of targeted, simple and easily accessible health information can be an important measure for improving reproductive health care, this article aims to evaluate the implementation of the PRENACEL program, a technological resources of communication in health by SMS, for the partner of pregnant women as a strategy to improve the participation and involvement of men in the PNC, as well as in the childbirth.

## Methods

### Design and setting

This study was a parallel, cluster randomized controlled trial comparing a routine prenatal care (PNC) with routine PNC plus PRENACEL. This study was conducted in Ribeirão Preto, a medium-sized city in the state of São Paulo, Brazil, which has 604,000 inhabitants described in the last national census. Regarding health, 59% of the municipality is covered by primary health care; it has five health districts and a total of 49 health care units [36–38].

The PRENACEL is a PNC communication strategy based on mobile phone short text messages (SMS). In this program, pregnant women and their partners receive educational messages related to pregnancy and childbirth. Twenty primary health care units (PHCUs) and four maternity hospitals took part of this study. The included health units were in the context of Brazilian Unified Health System (SUS – Sistema Único de Saúde), that provide health care totally free-of-charge.

**Fig. 1:**
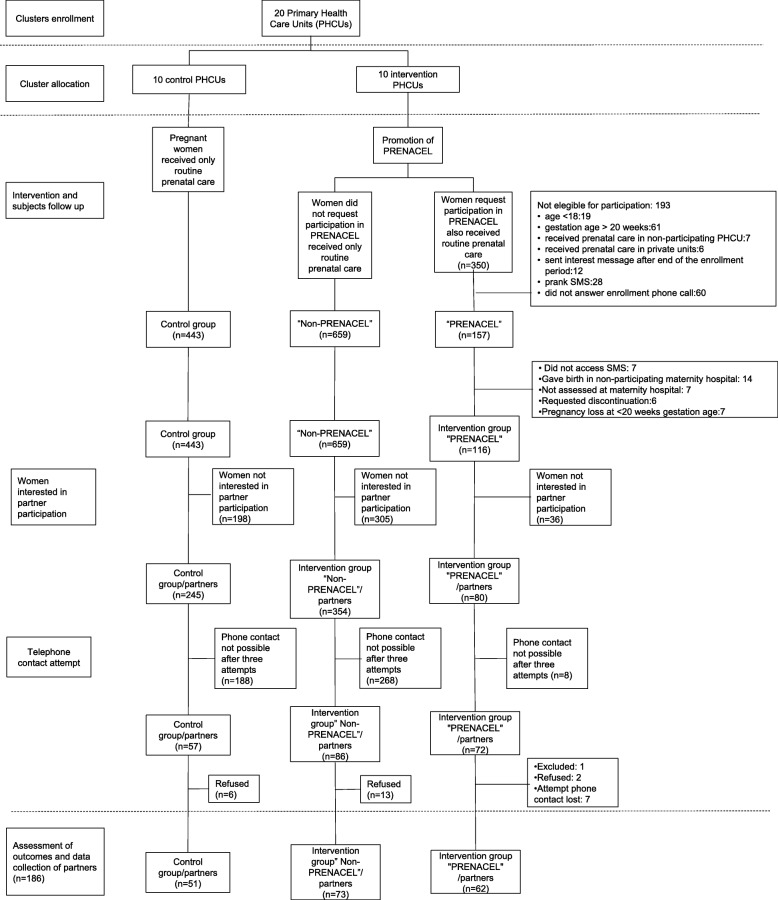
Study Flow diagram PRENACEL Partner

The study was implemented from April 2015 to March 2016. Women were passively recruited from intervention PHCUs through flyers and posters. Each woman who voluntarily registered in PRENACEL was invited to participate in the study and they were asked if they would want their partners to participate. Partner’s recruitment took place during a three-month period (April–June 2015). The Figure 1 illustrates the strategy and steps used for study recruitment: clusters enrollment, cluster allocation, intervention and subjects follow up, women interested in partner participation, telephone contact attempt and assessment of outcomes and data collection of partners. The partner’s interviews were carried out from September 2015 to March 2016.

### Ethical considerations

This study was carried out in accordance with the guidelines of Resolution 466/12 of the National Health Council and was approved by the Research Ethics Committee. All guidance regarding the study was provided to the interviewed partners by reading the consent form and subject acceptance was recorded, as the entire interview was recorded and informed consent was obtained for the study and subsequent publication. This study was registered at the Brazilian Clinical Trials Registry (REBEC, registration number RBR-54zf73, available at http://ensaiosclinicos.gov.br/rg/RBR-54zf73/. The component for pregnant women was published in 2017 and 2019 [39, 40].

### Study population

Cluster eligibility criteria: At cluster level, we selected the 20 PHCUs with largest number of women attending PNC in 2013 (number range from 116 to 569 pregnant women per PHCU per year, with an average of 205.05 pregnant women per year per PHCU). We selected all maternity hospitals that provided obstetric care free-of-charge within the Brazilian Unified Health System.

Woman eligibility criteria: The pregnant woman must be at least 18 years old; attended prenatal care in one of the 20 selected PHCUs; had gestational age of less than 20 weeks at the time of inclusion.

Partner eligibility criteria: The partner must be at least 18 years old; accompanied and attended PNC with his woman partner in one of the 20 selected PHCUs and the pregnant women had to agree to the partner’s participation and inform their contact.

Exclusion criteria: Partners and pregnant women who attended prenatal care at health units that were not selected for this study or who attended prenatal care at a private health care or in another city. Partner and the pregnant woman without access to a mobile phone.

### Data collection procedures

#### Intervention

The intervention was conducted in two levels: health unit and individual. At health unit level, the intervention was composed by three efforts: health care professionals participated in a workshop about PRENACEL; distributed flyers to women attending PNC and posters were displayed to invite pregnant women and partners to participate in the program. At individual level, the partners allowed by women to register at PRENACEL received a set of short messages via mobile phone and could also ask questions, send comments and concerns related to prenatal care. A total of 62 short text messages were used (Additional file 1: Table S1). The content of the messages received by the partner was related to the gestational age of each woman. All the participant partners received one or two messages per week during the period from five to 42 weeks of pregnancy and in the immediate postpartum.

These messages were based on the Mobile Alliance for Maternal Action (MAMA) [41] and adapted to the Brazilian health care system scenario. The adapted and translated messages were assessed by three health specialists, considering maternal health aspects. Finally, the messages were discussed by a focus group composed by male community health workers to adequate the wording of the messages for the target audience. Encourage partner to be involved in the woman pregnancy and postpartum were the main purpose of the PRENACEL Partner messages. This stage of the study that includes message adaptation is described in a published paper [42].

#### Control

In the control PHCUs, pregnant women and their partners received routine PNC. The partners of this group were only approached to participate in the study after childbirth (Fig. 1).

### Randomization

The cluster randomization of the 20 selected PHCUs was performed in two stages. In the first stage, two groups (with ten units each one) were randomly selected, balanced considering the size of the affiliated population and the vulnerability situation (evaluated by the number of beneficiaries of the government welfare program) of each health unit. Groups were balanced when the difference between population size and vulnerability was less than 15% stipulated by some criteria [39]. For the randomization, Microsoft Excel 2013 was used. The second stage consisted in the allocation of the PHCUs for intervention or control, with ten PHCUs each. This step was also performed by random draw using the randomization function.

### Blinding

The participants of PRENACEL, the PHCUs health professionals and the PRENACEL staff were not blinded to the intervention. Although, PHCUs health professionals not necessarily know the allocation status of each participant.

### Data management

PRENACEL data collection was described elsewhere [39]. PRENACEL Partner data was collected through telephone interviews with partners after childbirth. This process was conducted using the Skype® app to make the calls and recorded using the Kanda Callnote® app version 3.0.43. The voice recording process was used to store the spoke informed consent and to ensure the data quality with a second listening of each interview. The structured questionnaire (Additional file 2: Partner Questionnaire) was projected with the Research Electronic Data Capture (REDCap) platform [43]. The questionnaire had filters (filters, for example, prevented certain outliers) to avoid missing data or inconsistencies. Data was collected directly in electronic format.

### Sample size

To determine the sample size from our study, we based on two pieces of information: the sample size of pregnant women participating in the study and the percentage of men’s adherence to the partner prenatal program of the municipality. There was another line of research that was carried out with pregnant women and the calculation for the sample size of the primary study was determined to assess the effects of PRENACEL on prenatal care practices. Details of this calculation were previously published [39]. In summary, 581 women in the sample were required (145 pregnant women receiving the intervention and 436 in the control group) and the participation of 10 health units in each study group. And according to data from the municipal health department of the municipality, the percentage of adherence to the prenatal program of the partner that year was 37% [44].

Despite this information and the design of the study it was not possible to perform the necessary calculation, since our sample size depended on the acceptance of pregnant women in the participation of their partners. For this, we used a convenience sample. Based on the estimated sample of 145 women eligible for the PRENACEL program, the PRENACEL group would have 53 partners and the control group, 106, totaling 159 participants. The partners were divided into three groups: PRENACEL (partners who were users of the intervention health units, who received the messages via mobile phone), non-PRENACEL (partners that used the same health units, however, opted not to participate in the PRENACEL) and the control (partners who were users of the health units defined as control units).

### Statistical analysis

For the study, sociodemographic variables, such as age, marital status, race/color, years of schooling and education, work situation, economic class [45] and number of children, were identified. Were considered as the dependent variables: acceptance of the PRENACEL program, partner has attended at least one PNC consultations (nominal qualitative variable – yes or no), number of consultations that the partner attended (quantitative variable) and the presence of the partner at the moment of the childbirth (nominal qualitative variable - yes or no).

After cleaning the database, data were exported to Stata 9 for processing and analysis. The chi-squared test (χ2) and Fisher’s exact test were used as the statistical basis to calculate the associations between the independent and dependent variables between the groups. We analyzed the association by two models: Intention-to-treat (ITT) and per-protocol (PP). In ITT, we compared the PRENACEL group (who received the messages) plus the non-PRENACEL group (partners who did not receive the messages, but belonged to the intervention PHCUs) versus the control group. In PP, we compared the partners of PRENACEL group versus the control group. We also calculated the relative risk (RR) and confidence interval (CI 95%). To consider the association significant, a value of *p* < 0.05 or Fisher’s exact test < 0.05 was required.

## Results

A total of 186 partners were interviewed after intervention. Regarding the women who participated in the PRENACEL program (116 pregnant women); they were 62 partners (62/116; 53.4%) also participated plus 73 (73/354; 20.6%) in the non-PRENACEL group and 51 (51/245; 20.8%) in the control group. The average time between childbirth and the phone interview was 45 days (DP: 38 days; minimum of 3 and maximum 126 days). The Fig. 1 shows the study flow diagram.

Regarding the sociodemographic profile, an average age of 30 years (DP: 7.2 years; minimum of 19 and maximum of 54 years) was found, the majority of the interviewed men (95.2%) lived with their partner and 37.6% of them were legally married. The majority (51%) declared themselves brown. The interviewees had an average of 9.3 years of study, with almost 40% reporting that the last grade concluded was complete high school or incomplete higher education. Regarding the work situation, 93% were working during the interview period. In relation to the economic classification, 63.7% were classified in the C1/C2 group, which corresponds to a monthly mean household income of R$1446.24 to R$2409.01.^1^ Among the interviewees, the majority had two or more children and 62% already had a child with their current partner (Table 1).

**Table 1:** Sociodemographic characteristics and reproductive history of the partners interviewed, Ribeirão Preto

	Intervention Group		Control Group	
	PRENACEL	Non-PRENACEL		Total
Sociodemographic characteristics				
Age				
19–25 years	21 (33.9%)	27 (37.0%)	12 (23.5%)	60 (32.2%)
26–35 years	27 (43.5%)	33 (45.2%)	25 (49.0%)	85 (45.7%)
35–54 years	14 (22.6%)	13 (17.8%)	14 (27.5%)	41 (22.1%)
Total	62 (100.0%)	73 (100.0%)	51 (100.0%)	186 (100.0%)
Marital status				
Married or living together	57 (91.9%)	70 (95.9%)	50 (98.1%)	177 (95.2%)
Single or separated/divorced	5 (8.1%)	3 (4.1%)	1 (1.9%)	9 (4.8%)
Total	62 (100.0%)	73 (100.0%)	51 (100.0%)	186 (100.0%)
Race/color*1				
White	14 (22.9%)	24 (32.9%)	20 (39.2%)	58 (31.3%)
Brown	31 (50.8%)	41 (56.1%)	23 (45.1%)	95 (51.3%)
Black	12 (19.7%)	8 (11.0%)	7 (13.7%)	27 (14.6%)
Oriental	3 (4.9%)	0	1 (2.0%)	4 (2.2%)
Indigenous	1 (1.6%)	0	0	1 (0.5%)
Total	61 (100.0%)	73 (100.0%)	51 (100.0%)	185 (100.0%)
Years of study				
8 years or less	23 (37.1%)	29 (39.7%)	15 (29.4%)	67 (32%)
9 to 11 years	32 (51.6%)	29 (39.7%)	23 (45.1%)	84 (45.2%)
12 to 17 years	7 (11.3%)	15 (20.5%)	13 (25.5%)	35 (18.8%)
Total	62 (100.0%)	73 (100.0%)	51 (100.0%)	186 (100.0.%)
Education				
Illiterate or incomplete elementary education 1	2 (3.2%)	2 (2.7%)	2 (3.9%)	6 (3.2%)
Complete elementary education 1 or incomplete elementary 2	10 (16.1%)	16 (21.9%)	7 (13.7%)	33 (17.7%)
Complete elementary education 2 or incomplete high school	24 (38.7%)	26 (35.6%)	16 (31.4%)	66 (35.5%)
Complete high school or incomplete higher education	21 (33.9%)	29 (39.7%)	24 (47.1%)	74 (39.8%)
Complete higher education	5 (8.1%)	0 (0.0%)	2 (3.9%)	7 (3.8%)
Total	62 (100%)	73 (100%)	51 (100%)	186 (100%)
Paid work				
Yes	58 (93.5%)	68 (93.1%)	47 (92.1%)	173 (93.0%)
No	4 (6.5%)	5 (6.9%)	4 (7.9%)	13 (7.0%)
Total	62 (100.0%)	73 (100.0%)	51 (100.0%)	186 (100.0%)
Social class*2				
A/B1-B2	18 (30.5%)	11 (15.5%)	13 (26.5%)	42 (23.5%)
C1- C2	34 (57.6%)	47 (66.2%)	33 (67.4%)	114 (63.7%)
D-E	7 (11.9%)	13 (18.3%)	3 (6.1%)	23 (12.8%)
Total	59 (100.0%)	71 (100.0%)	49 (100.0%)	179 (100.0%)
Reproductive history				
Number of children				
0 or 1	34 (54.8%)	35 (47.9%)	22 (43.1%)	91 (48.9%)
2 or more	28 (45.2%)	38 (52.1%)	29 (56.9%)	95 (51.1%)
Total	62 (100.0%)	73 (100.0%)	51 (100.0%)	186 (100.0%)
Number of children with current partner				
0 or 1	40 (64.5%)	43 (58.9%)	34 (66.7%)	117 (62.9%)
2 or more	22 (35.5%)	30 (41.1%)	17 (33.3%)	69 (37.1%)
Total	62 (100.0%)	73 (100.0%)	51 (100.0%)	186 (100.0%)

*1 missing value = 1

*2 missing value = 7

In the descriptive analysis of all participating partners (186), 82.8% of them attended their companion’s PNC and the majority of them (68.2%) reported having attended one and up to five PNC consultations.

Regarding the analysis made between the groups, there was no statistically significant difference when we compared whether or not partners participated in PNC, either by ITT or PP. However, there was a statistically significant association with PP when assessing the number of PNC consultations and the presence at birth, which showed that PRENACEL partners, when they participated, go on a larger number of PNC consultations and were more present at birth (*p =* 0.02 and *p =* 0.01 respectively). The RR and CI 95% confirmed these findings (Table 2).

**Table 2:** Frequency of the outcomes of partner attended at least one PNC consultation, number of consultations the partner attended and presence of the partner at the birth according to the group; *p*-value of the chi-squared test and Fisher’s exact test, relative risk (RR) and confidence interval (CI 95%), Ribeirão Preto

Variable	Intervention group			Control	*p (ITT)	RR (ITT) (CI 95%)	*p (PP)	RR (PP) (CI 95%)
	PRENACEL	Non-PRENACEL	All intervention group					
Partner attended at least 1 PNC consultation								
Yes	50 (80.6%)	59 (80.8%)	109 (80.7%)	45 (88.2%)	0.227	0.90 (0.22–1.45)	0.273	0.90 (0.19–1.6)
No	12 (19.4%)	14 (19.1%)	26 (19.3%)	6 (11.8%)				
Total	62 (100%)	73 (100%)	135 (100%)	51 (100%)				
Number of consultations								
6 or more	19 (38%)	16 (27.1%)	35 (32.1%)	8 (17.8%)	0.073	1.8 (0.92–5.21)	0.020	2.27 (1.17–8.09)
1 to 5	27 (54%)	43 (72.9%)	70 (64.2%)	35 (77.8%)				
Missing value	4 (8%)	0 (0.0%)	4 (3.7%)	2 (4.4%)				
Total	50 (100%)	9 (100%)	109 (100%)	45 (100%)				
Presence at the birth								
Yes	46 (74.2%)	43 (58.9%)	89 (65.9%)	27 (52.9%)	0.09	1.26 (0.91–3.39)	0.013	1.44 (1.22–6.07)
No	15 (24.2%)	30 (41.1%)	45 (33.3%)	24 (47.1%)				
Missing value	1 (1.6%)	0 (0.0%)	1 (0.8%)	0 (0.0%)				
Total	62 (100%)	73 (100%)	135 (100%)	51 (100%)				

*p < 0,05

We also calculated the RR and CI 95% between PRENACEL and non-PRENACEL groups (Intervention group). In the variable number of consultations that partners participated it was found a RR = 1.52 and CI = 0.89–2.62 and in the variable presence at the birth a RR = 1.28 and CI = 1.01–1.36. In the comparison of PRENACEL group with non-PRENACEL group it seems that there was a trend of the first group to attend more consultations and to be more present at the time of birth (results not shown in the table).

## Discussion

Our findings suggest that partners that received SMS messages related to PNC were more frequently present at birth, favoring childbirth companionship. We also observed a trend towards increased frequency of partner in PNC consultations through the messages strategy. The large majority of the participating partners attended at least one PNC consultation, despite the recent inclusion of the partner in PNC as a program in the PHCUs and still in the process of being implemented by the municipalities in Brazil.

Narrowing the understanding of pregnancy to a merely biological process leads to the understanding that PNC should be limited to the presence of women. This understanding probably means a number of limitations, such as the low presence of men in the health service, since many of them do not feel any identification with the services, the unfavorable hours for the consultations and the mistreatment experienced by the couple regarding the care of the team. The lack of space to accommodate the companion and the lack of adequate local infrastructure to attend PNC consultations generate discomfort and further distance the partners. These are some of the difficulties that need to be overcome to ensure the partner’s involvement of the PNC [23, 46].

Similar studies have shown that health communication technologies had a good acceptance and the messages were considered useful as a form of support for the new fathers and they attract men to a closer experience in the care and support of their partner during the pregnancy [26, 47]. Our results show good acceptability of this program, a little more than half of the partners participated, comparing to data of partner’s participation in PNC from the local SUS which in 2014 was 37% [44].

Fletcher et al. (2016) identified that intervention for fathers also using SMS messages from the prenatal to the postnatal period would be a good strategy. And that replies from partners suggest that the messages were relevant and useful, but that the 4Dad program had modest adherence [48]. The same author in another study showed the importance of providing a parent-specific program that helps support men’s role as parents. And the importance of using an interactive health education process, such as mHealth, where technological change is rapid and must be flexible to the proposed objectives. And it points to the benefits of mobile health programs for prospective parents by considering them as structural and individual processes that combine to achieve targeted outcomes, in this case targeted at the mental health of partners, and to assist in the transition to parenthood [49].

Some authors have studied the applicability and effectiveness of health education programs using eHealth to improve the knowledge of future parents in the pregnancy, the birth, the postpartum period and breastfeeding, encouraging men to become involved in the pregnancy of their partner [29, 50–53]. However, the majority of these programs were focused on pregnant women, making it difficult for men, who already have less information, to obtain knowledge about the pregnancy [54].

Regarding the participation of PNC consultations our result corroborates data from the Brazilian Ministry of Health, which showed that 80% of the men followed the prenatal care of their partners [10].

A national campaign was carried out in Indonesia as a strategy to encourage partners to attend prenatal consultations with their partners. The men received information about the signs of risk during pregnancy and childbirth as one of the ways to ensure safe motherhood. Among the respondents, 92% of the men categorized as trained according to the campaign participated in the prenatal consultations, while 81% of the men of the untrained group participated in the consultations [55]. Although these types of programs are useful tools to stimulate male participation there are some studies showing the decrease of father interest in prenatal consultations after the program.

Contrary to expectations, Berti et al. (2015) hypothesized that these programs could provide greater autonomy for the women, culminating in a decrease in the involvement of the fathers [56].

Regarding the participation during the labour, of the total number of women who completed the international survey “Listen to the mothers III” almost all (99%) had some support during the birth, which was most often provided by the husband or partner (77%) [57, 58].

The national survey “*Nascer no Brasil*” showed that 75.5% of the women had a person accompanying them during the hospitalization, however, only 18.8% had a person accompanying them at all time. Although the partner was mentioned by just over a third of the study sample (35.4%) as the companion of choice for the woman, the partner was chosen in the majority of cases compared to other companions. In addition, 84.5% of the women reported that the presence of a companion helped a lot for them to have a good birth, according to their right guaranteed by the law of the companion (Law No. 11.108, 2005) [59].

Men are not easily accessible for research interview and often the best place to find them is in the workplace. Therefore, to obtain a greater number of interviews we choose to carry them out via telephone in the evening period by Skype® application. Since many of the interviewees resided in more distant places or neighborhoods sometimes the interview was interrupted due to signal failures. When this happened the contact was made again, but it can be considered a limitation of the study.

Another limitation of the study was the calculation and the small number of the sample size which makes the findings stay further from the truth in the population, a source of imprecision. Thus we can not infer about the findings from a population-wide perspective or make generalizations. But we calculated the sample size by convenience, according to the acceptance of pregnant women in the participation of their partners and this was a pilot study to prepare a larger study based on experience, limitations and thinking of improvements on a larger scale.

But as strengths we can indicate that we respect the choice of women, asking them first if they wanted the partners to participate in the program. This concerns issues related to gender and power spaces. Environments related to prenatal and childbirth are spaces with a greater female presence, which could characterize a more welcoming environment the woman in which she could be protagonist of their actions. And to have a man in this place without the consent of the woman who could be more space for man to assert himself and try to impose his desires would not be respecting the issues related to the gender.

And in our evaluation, this study was pioneer in our country and the strategy using SMS seems to be as a way to improve and encourage the participation of the partner in PNC consultations and childbirth.

## Conclusion

A SMS program, as PRENACEL, for PNC is a useful supplement contributing to increase labour companionship at childbirth. This program worked with a soft technology, easy to implement and relatively inexpensive, which could contribute to improving maternal health.

The results show that a health education strategy using communication technology seems to have good acceptability and a very promising role in engaging men in the prenatal care, birth and postpartum care of their female partners.

## Supplementary information

**Additional file 1.** Messages sent to partners.

**Additional file 2.** Partner Questionnaire.

## Data Availability

The materials as well as the data of this research are available in a database recorded in excel and online version for review that requested upon plausible recommendation.

## Abbreviations

CCEB: Brazil Criteria for Economic Classification – Critério de Classificação Econômica Brasil
CHW: Community health workers
MAMA: Mobile Alliance for Maternal Action
MMR: Maternal mortality ratio
PHCUs: Primary health care units
PHUs or PDHUs: Primary and District Health Units
PNC: Prenatal care
REBEC: Brazilian clinical trials registry
SDGs: Sustainable development goals
SMS: Short message service
SUS: National Health System - Sistema Único de Saúde
WHO: World Health Organization

## Footnotes

1 Equivalent of USD 359.76 to USD 599.25 (Conversion rate of 1.00 USD = 4,02 BRL)
