# Supplementary material for: PRENACEL partner - use of short message service (SMS) to encourage male involvement in prenatal care: a cluster randomized trial
Source: Reprod Health. 2020 Apr 6;17:45. doi: 10.1186/s12978-020-0859-6 (PMC7132868; doi:10.1186/s12978-020-0859-6)
Supplement: Supplementary file 1 — Additional file 1. Messages sent to partners. [file 12978_2020_859_MOESM1_ESM.pdf]

Table 1

PARTNER MESSAGE	GESTATIONAL WEEK
Congratulations, there is a baby on the way! Encourage your partner to go to prenatal consultations and join you too!	5
Remind your pregnant partner to take their ferrous sulfate and folic acid tablets. She can get the pills at the health unit.	6
It is important for pregnant women to stay healthy. Check if your mate is eating well, eating meat, beans, fruits and vegetables.	7
Make sure everyone in the family wash their hands after using the bathroom and before meals. This helps keep your mate healthy.	8
Reassure your partner that nausea in early pregnancy is common. Cornstarch crackers and ginger or lemon tea may help.	8
It is important to begin preparation for childbirth from the beginning of pregnancy. Mind and body together to bring a healthy baby to this world.	9
Have you started prenatal care yet? At health unit your partner will perform tests, receive ferrous sulfate tablets and folic acid and update vaccines.	9
Pregnant women with the flu can get very sick. Make sure the pregnant woman has already had the flu vaccine.	10
The pregnant woman's body is prepared for normal birth. Labor is a sign that your baby is ready to be born.	10
Minor bleeding may occur during pregnancy. In this case, seek medical attention so as not to lose the baby.	11
See if your partner drinks plenty of water and feeds properly. This will keep her strong and healthy.	11
Most women prefer normal birth in early pregnancy. Talk to your partner that normal birth is safer. She will make it!	12
Having a companion during childbirth will make your partner feel safer. It is your right!	12

Talk to your partner about who will accompany you during childbirth. Whoever it is, there needs to be someone willing to help her.	13
Many women are convinced by acquaintances, family and even health professionals that caesarean section is better. Believe me: normal birth is better!	13
Make sure your mate is not eating weird things like earth. If she wants to eat these things, take her to the health unit.	14
In some situations a caesarean section may be required. If this happens, trust maternity health professionals.	14
Encourage your partner not to smoke and not to drink alcohol. This is bad for the baby. Ask smokers who smoke outside the home and away from the pregnant woman.	15
Keep your mate healthy by ensuring that she eats only fresh, well-cooked food. Food out of the fridge can spoil and harm the pregnant woman.	16
Accompanying your partner during labor can help her better withstand contractions.	17
Being pregnant makes your partner tired. Have other family members help your partner and give her time to rest.	17
Help your partner walk and seek a more comfortable position during labor. It helps her.	18
Help your mate get well, encourage her to rest from time to time. This makes her stay healthy.	18
The pregnant woman's diet should have 3 main meals and 3 healthy snacks. Small portions at a time and more often is the way to less discomfort.	19
Syphilis is a sexually transmitted disease that can make the baby very sick. In prenatal care you can take exams to see if you have syphilis or not.	19
Syphilis? It is important to treat with penicillin injections and to use condoms. Be sure to do the complete treatment!	20

Does your mate already feel the baby move? Encourage her to follow the baby's movements and look for the health unit if the movements slow or stop.	20
If swelling of the hands, feet and face increases sharply, it may be a sign of trouble. Take your partner to the health unit if you notice this change.	21
Vomiting, fever, pain, colic, and bleeding are signs of trouble. Take the pregnant woman to the health unit if any of these signs appear.	21
Many women prefer to give birth sitting, on all fours, or squatting. Your partner can choose the most comfortable position.	22
Use massage to relieve your partner's back discomfort. Pillows to support her back when she lies down help make her more comfortable.	22
You can help your partner in motherhood. Help her by giving support, caring, encouragement, massage, and helping her in whatever she can.	23
Make sure your mate is going to prenatal care. Try to save some money because the arrival of a child always generates additional expenses.	24
Syphilis? Take the test, use a condom and, if positive, complete the treatment with all injections. This helps your baby be born healthy.	24
If your mate has swollen feet, ask her to rest with them up. In this case it is important to measure the pressure in the health unit.	25
Diabetes can happen in pregnancy. It causes: tiredness, too much thirst, too much pee and weakness. Take your mate to the health unit if in doubt.	26
Walking, crouching, moving the hips, massaging, bathing and seeking a more comfortable position can help your partner have less pain during childbirth.	27
In labor, your mate will have uncomfortable, often painful contractions. Between contractions she can rest and have her	28

support at this time.	
Assure your mate that the baby turns when she is ready. Labor is the sign that the time for a baby has come.	29
During labor, you can help your partner with back massage by giving her fluids and helping her find a comfortable position.	29
During labor, your partner can walk, bathe, seek a more comfortable position and receive massages. It relieves the pain!	30
Do you already know how to get to the maternity ward where your partner will have the baby? Get organized to take it when the time comes.	30
Take your mate to maternity when she goes into labor. Strong and regular contractions or if the bag bursts are signs that the time has come.	31
A trusted companion during childbirth will make your partner more relaxed. This makes labor faster.	31
During normal labor, your partner can also drink fluids freely and eat light foods.	32
Will you accompany your partner during childbirth? It is your right!	32
Encourage your partner to breastfeed as soon as she gives birth. This first milk and the contact with your mate's skin is very good for your baby.	33
Your mate's body is prepared for normal birth. She doesn't need fasting, vein serum, or artificial oxytocin at first.	34
Scheduled Caesarea before labor can give birth to your baby before it is ready. Labor is a sign that the time has come!	35
You and your family should be aware of danger signs: fever, bleeding, seizures, difficulty breathing. Seek help quickly if something like this occurs.	35
Normal childbirth does not let your partner loose, nor does her bladder fall. Caesarean section has more risks for her and her	36

baby.	
Have you ever visited the maternity ward? Do you know how they will get there? Now it's time to know all the details.	36
Talk to the doctor about childbirth. Have you ever heard that some women get a cut in the vagina during childbirth? Most of the time it is not necessary.	37
Be kind and polite to the maternity staff, but make your partner's preferences clear!	37
Labor is a sign that the baby is ready to be born. You can help by encouraging your partner to take short walks.	38
Observe the movements of your baby. If they slow down or stop, look for motherhood. Your baby needs to be evaluated.	38
Support your partner's initiative to breastfeed as soon as she gives birth. Breast milk and contact with your partner's skin are good for your baby.	39
Ask your partner to observe the baby's movements. If they slow down or stop, look for motherhood. The baby needs to be evaluated.	39
The moment of birth is coming! Watch for warning signs such as contractions, loss of fluid or blood, and poor baby movement.	40
Calm down! The baby knows the right time to be born. Observe the warning signs: loss of blood or fluid, stopping baby movements.	41
If your partner has not yet gone into labor this week, look for the maternity ward or health unit. She and the baby need to be evaluated	42
Our journey together ends here. We wish you and your family great strength and the best of luck in the world.	42
