# Supplementary material for: PRENACEL partner - use of short message service (SMS) to encourage male involvement in prenatal care: a cluster randomized trial
Source: Reprod Health. 2020 Apr 6;17:45. doi: 10.1186/s12978-020-0859-6 (PMC7132868; doi:10.1186/s12978-020-0859-6)
Supplement: Supplementary file 2 — Additional file 2. Partner Questionnaire. [file 12978_2020_859_MOESM2_ESM.docx]

**Supplementary Material - Partner Questionnaire**
